# Economic Ripple Effects of Individual Disasters and Disaster Clusters

**DOI:** 10.1007/s13753-022-00451-0

**Published:** 2022-11-25

**Authors:** Zhengtao Zhang, Ning Li, Ming Wang, Kai Liu, Chengfang Huang, Linmei Zhuang, Fenggui Liu

**Affiliations:** 1 Key Laboratory of Environmental Change and Natural Disaster, Ministry of Education, Beijing Normal University, Beijing, 100875 China; 2 Academy of Disaster Reduction and Emergency Management, Ministry of Emergency Management and Ministry of Education, Beijing Normal University, Beijing, 100875 China; 3 State Key Laboratory of Earth Surface Processes and Resource Ecology, Beijing Normal University, Beijing, 100875 China; 4 Faculty of Geographical Science, Beijing Normal University, Beijing, 100875 China; 5 School of National Safety and Emergency Management, Beijing Normal University, Beijing, 100875 China; 6 Academy of Plateau Science and Sustainability, Xining, 810016 China

**Keywords:** Disaster clusters, Disaster risk management, Economic ripple effects, Indirect economic losses, Input-output model

## Abstract

Disaster clusters refer to major disasters that cluster in space and time without any linkage, resulting in large direct damage and economic ripple effects (EREs). However, the cumulative EREs caused by a disaster cluster may not be equal to the summation EREs of the individual disasters within a cluster. We constructed a global economic ripple input-output model suitable for the analysis of disaster clusters and demonstrated the extent of this difference with the example of two typical catastrophes that occurred in 2011 (the Great East Japan Earthquake and the Great Thailand Flood), within an interval of only 136 days. The results indicate that: (1) The EREs suffered by 11 of the 35 countries affected (30%) are “1 + 1 > 2”, and “1 + 1 < 2” for 24 of the 35 countries affected (70%). This indicates that there is a significant difference between the cumulative and the summation losses. The difference is related to factors such as trade distance, economic influence of disaster-affected sectors, and trade ties; (2) The EREs are more than two times the direct loss and have an industrial dependence, mostly aggregated in key sectors with strong industrial influence and fast trade times in the industrial chain; and (3) Additional EREs due to the extension of the recovery period will be aggregated in countries with close trade ties to the disaster-affected country, further magnifying the difference.

## Introduction

**Fig. 1:**
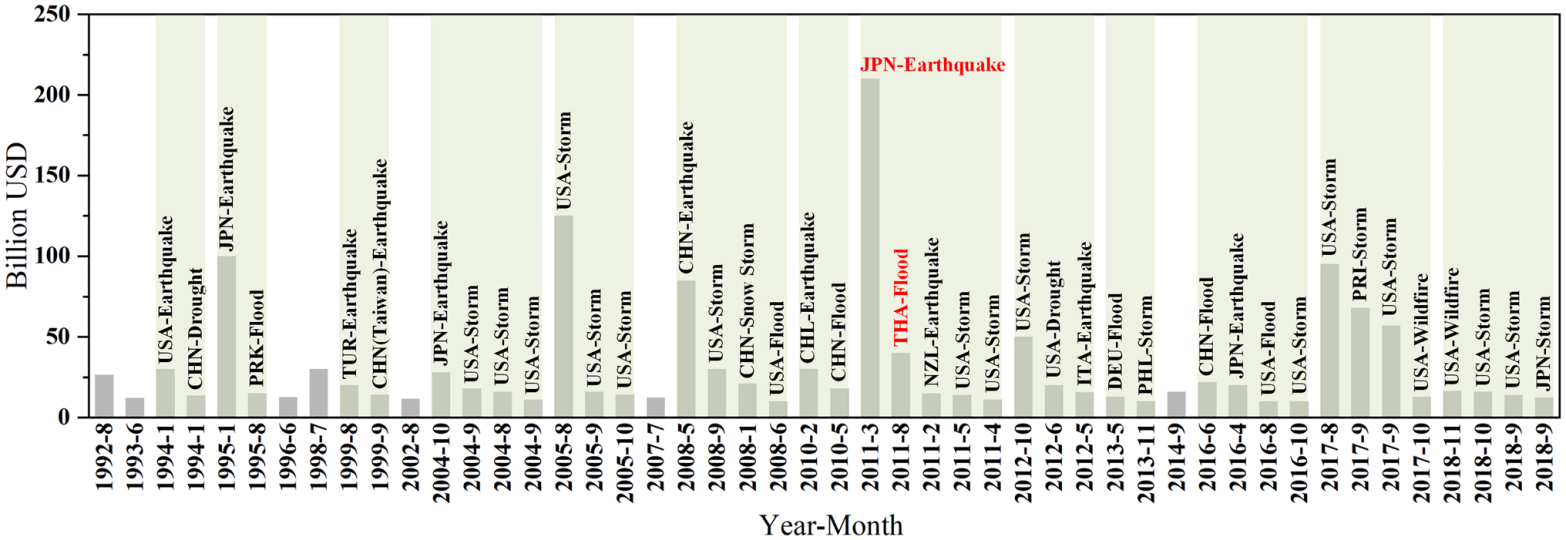
Disasters with direct economic loss greater than USD 1 Billion, 1992−2018. Green blocks indicate catastrophic disasters that occurred in the same year. *Data source* CRED (2021)

The frequency and intensity of natural hazards and disasters are on the rise and among them, “catastrophes” have received the most attention because they not only cause great damage to people’s lives and property, but also have serious impacts on the regional, national, and even global economy (Kuppusamy 2008; Liu et al. 2018; Prihantini 2020; Cook et al. 2021). If a catastrophe is defined by the standard that the direct economic loss of a single disaster exceeds USD 1 billion, by analyzing the types and frequency of catastrophes that occurred around the world from 1992 to 2018 (CRED 2021), we found that these events mainly included earthquakes (including tsunamis), typhoons, and floods, and the frequency of catastrophes showed an increasing trend (Fig. 1). The proportion of years when two or more catastrophes occurred in the same year between 1992 and 2018 is over 50%. Different from the disaster chain, these catastrophes barely recur in the same location and some of them occur in countries far apart, but they occur at short intervals and there is no causal relationship between them (Fig. 1). According to the classification of disaster types based on catastrophology (Saarinen et al. 1973; Shi et al. 2014), these events are called “disaster clusters” in our study. Disaster cluster refers to the simultaneous or successive occurrence of catastrophic disasters in a specific period of time, and there is no causal relationship between them. With the aggravation of global climate change, population growth, and global wealth increase, the occurrence of disaster clusters has become increasingly frequent (Fig. 1).

Although there have been extensive studies on the formation mechanisms, economic losses, and risk prevention of catastrophic disasters (Ward et al. 2013; Winsemius et al. 2013; Li et al. 2014; Winsemius et al. 2015; Tokui et al. 2017), there are few targeted studies on the losses caused by disaster clusters and their prevention (Okazumi and Nakasu 2015; Wang et al. 2021). The impact of a catastrophe has the characteristics of a large direct economic loss (DEL) and long post-disaster recovery period (Hallegatte 2008). The larger DEL will lead to more serious production shutdowns or reductions, labor interruption, industrial chain disruption, and other problems in the longer post-disaster period. These problems will trigger supply and demand imbalance in the global industrial chain, resulting in large indirect economic loss (IEL) in the disaster area and economic ripple effects (EREs) in countries around the world. The IEL and EREs may be even bigger than the DEL (Wu et al. 2011; Zhang et al. 2018; Koks et al. 2019), and are not less important than the DEL (Xia et al. 2016; Zeng et al. 2019).

The above conclusions have been empirically tested (Rose and Liao 2005; Hallegatte 2014; Koks et al. 2015). The EREs in other countries/regions caused by catastrophe through the global supply chain have received attention since the 2011 Great East Japan Earthquake (Park et al. 2013; Arto et al. 2015), and have been extensively studied since the COVID-19 pandemic (Guan et al. 2020; Davlasheridze et al. 2021; Verschuur et al. 2021). Due to the spatial independence of catastrophes in a disaster cluster, the assessment of the DELs from a disaster cluster uses the accumulation of the DELs of the individual catastrophes. The assessment of the EREs caused by the DELs also has depended on the independent assessment of the individual catastrophes (Chhibber and Laajaj 2008). Nonetheless, because the global industrial chain can connect products and services across industries in different countries/regions, the impact of a disaster cluster on the global industrial chain is integrated rather than an arithmetic summation (Zeng and Guan 2020). If the occurrence of the second catastrophe is still within the recovery period of the previous catastrophe, the economic system will be attacked again when it has not fully recovered from the previous disaster shock, even if the second catastrophe occurs in another location. In this case, an important scientific question is: does the ERE have a cumulative effect? Is the ERE generated by the disaster cluster in countries around the world equal to the sum of the EREs caused by each of the catastrophes?

This study selected the 11 March 2011 Great East Japan Earthquake (hereafter referred to as “Japan Earthquake” or “JE”) and the 25 July 2011 Great Thailand Flood (hereafter referred to as “Thailand Flood” or “TF”) as a disaster cluster to examine the cumulative effect of the EREs. The Thailand Flood that covered Thailand’s largest national industrial park and the Japan Earthquake that caused a tsunami and electricity failure both directly led to huge capital damage and labor and business interruptions for Japan’s and Thailand’s automobile, electrical equipment, and other manufacturers. The two catastrophes were only 136 days apart. Because Japan’s and Thailand’s manufacturing industries play an important role in global supply chains, the impact of the catastrophes on these industries also had EREs on the global economy. For example, many well-known hard disk factories in the national industrial parks of Thailand were unable to meet the global products demand, which resulted in a 40% or so increase in the global hard disk cost per GB in the two quarters after the Thailand Flood, and the price of a 500 GB hard disk in Changsha, China doubled within one month. The impacts on the automobile and cosmetic industries of Japan caused by the Japan Earthquake had a similar ripple effects on the global supply chain. However, most of the studies have focused on assessing the EREs of the two catastrophes independently based on the global industrial chain, ignoring the cumulative effect of EREs caused by the disaster cluster composed of these two disasters (Kajitani and Tatano 2014; Higashi 2020; Tanoue et al. 2020; Yagi et al. 2020).

Our study constructed a dynamic assessment model to evaluate the cumulative effect of IELs in Thailand and Japan, and the cumulative effect of EREs in 36 countries/regions in the world caused by the disaster cluster. By comparing and analyzing the difference between the cumulative effect and the summation result of IEL/ERE, we reveal the amplification effect of disaster clusters on global EREs, explore the key factors behind this effect, and provide quantitative answers to the question of whether “1 + 1 = 2.” The study emphasizes the importance of the EREs caused by disaster clusters, providing a theoretical basis and methodological support for governments to prevent the major economic risks of global catastrophes.

## Research Method and Scenario Setting

This section describes the algorithm flow of the global economic ripple input-output (GERIO) model, the key equations and the data sources of key evaluation parameters and variables, and the uncertainty analysis of the evaluation results.

### Data

**Fig. 2:**
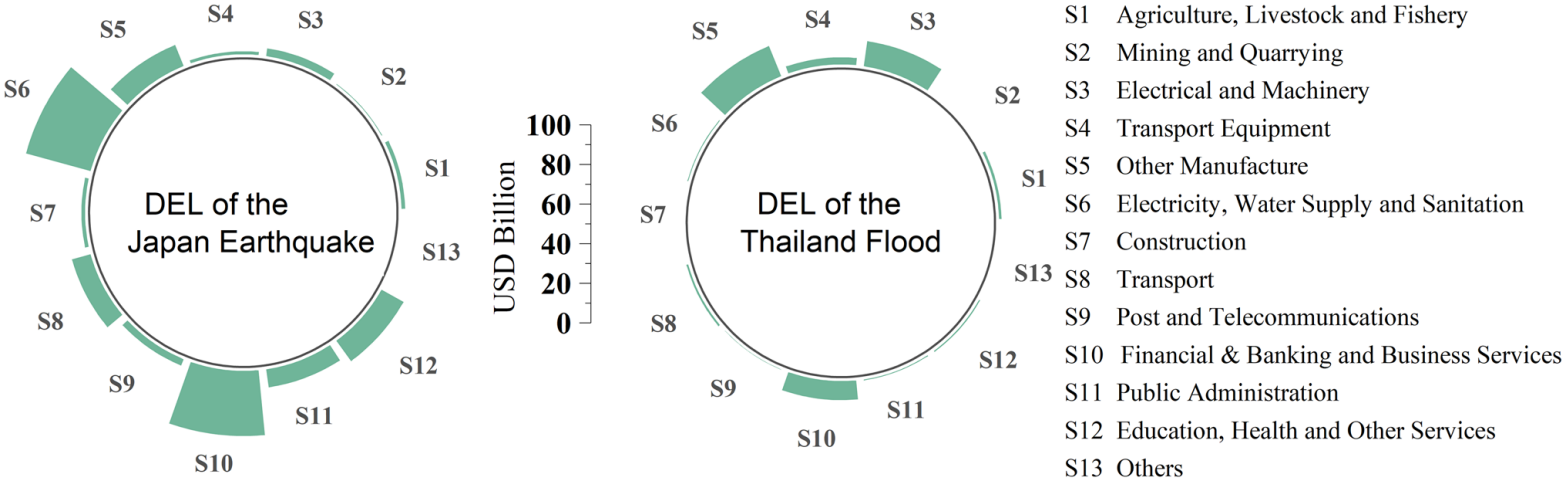
Direct economic losses (DELs) of 13 sectors of Japan caused by the 2011 Japan Earthquake (JE), and of Thailand caused by the 2011 Thailand Flood (TF)

In order to evaluate the EREs of the JE and TF on countries on all continents, we used the 2010 world input-output table derived from the Eora database (Lenzen et al. 2012; Lenzen et al. 2013), which covers 26 sectors in 189 countries/regions. Considering the differences between developed and developing countries, the trade relations with Thailand and Japan, continent location, the complexity of the model evaluation, and to balance the statistical deviations of the Eora input-output table, we selected the following 37 typical countries/regions and combined the others into a region (Rest of the world, RoW): Japan (JPN), Thailand (THA), Malaysia (MYS), China (CHN), Indonesia (IDN), Korea (KOR), United States (USA), Saudi Arabia (SAU), Australia (AUS), United Arab Emirates (ARE), Singapore (SGP), France (FRA), Germany (DEU), Russian Federation (RUS), Egypt (EGY), Canada (CAN), India (IND), Spain (SEP), Brazil (BRA), Iran (IRN), South Africa (ZAF), Myanmar (MMR), Czech Republic (CZE), Turkey (TUR), Poland (POL), Argentina (ARG), Vietnam (VNM), Yemen (YEM), Kazakhstan (KAZ), Cambodia (KHM), Pakistan (PAK), Libyan Arab Jamahiriya (LBY), Turkmenistan (TKM), Congo (COD), Mongolia (MNG), Kyrgyzstan (KGZ), and Tajikistan (TJK). In addition, the 26 original sectors were merged into the following 13 sectors based on United Nations (2008): Agriculture, Livestock and Fishery (S1); Mining and Quarrying (S2); Electrical and Machinery (S3); Transport Equipment (S4); Other Manufacture (S5); Electricity, Water Supply and Sanitation (S6); Construction (S7); Transport (S8); Post and Telecommunications (S9); Financial & Banking and Business Services (S10); Public Administration (S11); Education, Health and Other Services (S12), and Others (S13). The data for the DELs (Fig. 2) and number/times of people affected by the 2011 Great East Japan Earthquake and the 2011 Great Thailand Flood and the basic socioeconomic data (such as GDP, working population by industry, and trade time) come from World Bank reports (World Bank 2012; 2021), official websites (RIETI 2010; NESDC 2021; Reconstruction Agency 2021; Statistics Bureau of Japan 2021), and literature review (Mayer and Zignago 2011; Okazumi and Nakasu 2015; Poaponsakorn and Meethom 2015; Tokui et al. 2017).

### Comparative Scenario Settings

In order to examine the difference between the cumulative effect and summation result of IEL/ERE, we set three comparative scenarios (Table 1): (1) The Japan Earthquake occurred but the Thailand Flood did not; (2) The Thailand Flood occurred but the Japan Earthquake did not; (3) Both the Japan Earthquake and the Thailand Flood occurred. Then, we evaluated the losses based on the model under each scenario.

**Table 1:** Three comparative scenarios of two catastrophes—the 2011 Japan Earthquake (JE) and the 2011 Thailand Flood (TF)—that occurred independently, and the disaster cluster of the two (JE and TF)

ID	Scenario description
Scenario 1	Evaluate the indirect economic loss (IEL) caused by JE in Japan independently and the economic ripple effect (ERE) caused by JE in all countries (including Thailand) in the world. Assume that the TF did not occur
Scenario 2	Evaluate the IEL caused by TF in Thailand independently and the ERE caused by TF in all countries (including Japan) in the world. Assume that the JE did not occur
Scenario 3	Evaluate the IELs in Japan and Thailand caused by JE and TF, and the EREs in countries (including Japan and Thailand) around the world. This is an actual scenario

### Methodology

**Fig. 3:**
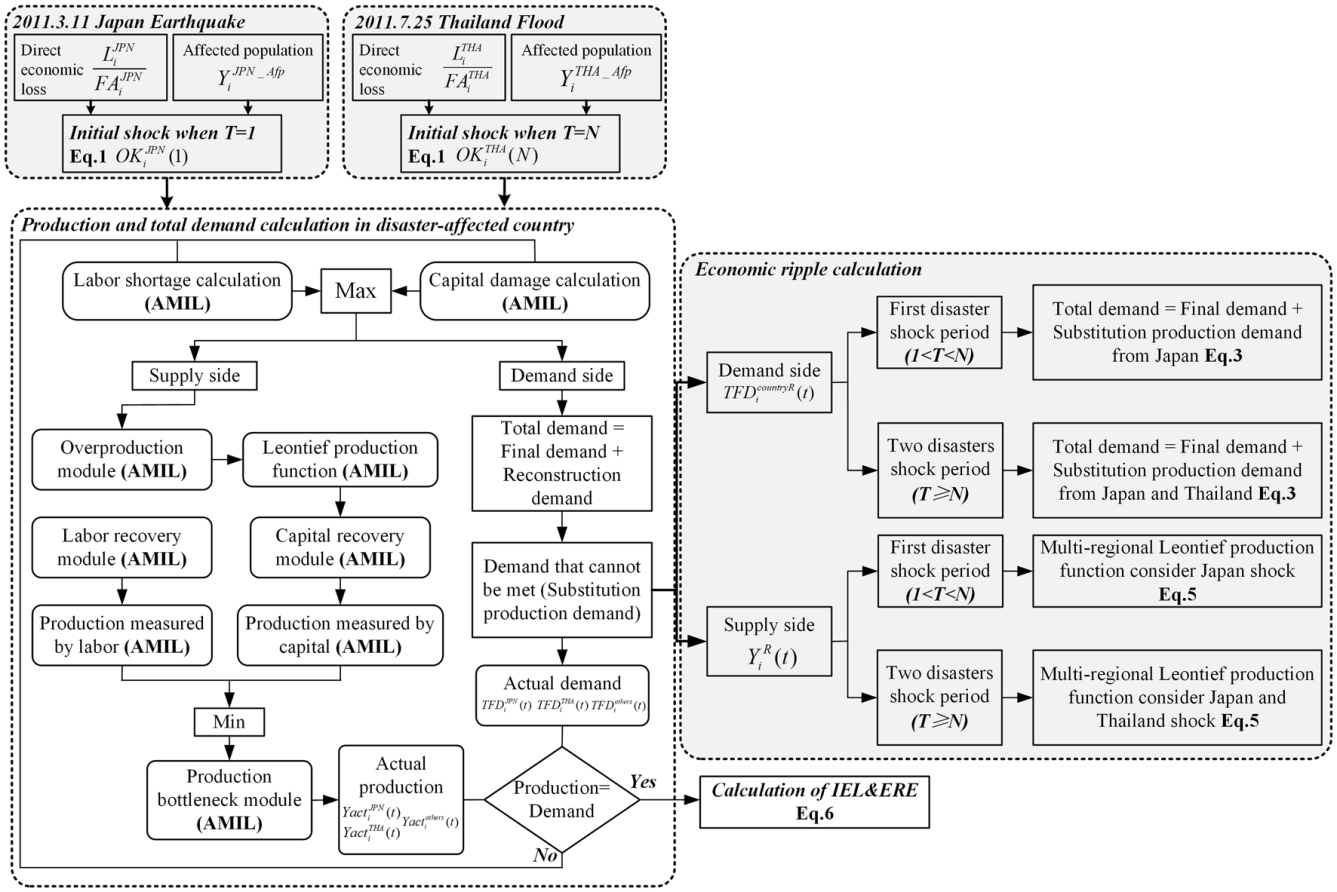
Flow diagram of the global economic ripple input-output (GERIO) model

The GERIO model used in this study was derived from the Adaptive Multi-regional Input-output with Labour (AMIL) model (Zhang et al. 2019). The model can integrate the impact of the capital stock damage and the affected population on the economic system during the recovery period and evaluate the economic ripple effect outside the disaster area on the supply and demand side (Zhang et al. 2018; Zhang et al. 2019). However, the AMIL model cannot evaluate the sequential impact of the disaster cluster on the global industrial chain. Our study makes a targeted optimization of the economic ripple calculation module so that it can evaluate the change characteristics of the sequential shocks for the economic system and the EREs on the regions outside the disaster area. The flow diagram of the model is shown in Fig. 3.

The GERIO model follows the AMIL model’s assumption that there is still a rigidity linkage among sectors and no substitution product across sectors in the disaster-affected country. However, in order to evaluate the ERE between countries, the model adds an algorithm for the substitution production outside the disaster-affected country for unmet recovery demand, and evaluates the impact of supply reduction and demand increase on the global industrial linkage from both the supply and demand sides.

The model evaluates the impact of catastrophe shocks on production capacity and total demand. It first introduces a variable *OK* to characterize the supply-demand imbalance in the disaster-affected countries (Japan and Thailand)—from the initial shock moment to the recovery period—due to the Japan Earthquake and the Thailand Flood. The time of the Japan Earthquake is set as *t *= 1, and that of the Thailand Flood is set as *t = N *(*N = 136*); $OK_{i}^{JPN}$ and $OK_{i}^{THA}$ (Eq. 1) represent the extent of the actual supply-demand imbalance of the *i*-th sector of Japan and Thailand, respectively:

1 $$\left\{ \begin{gathered} OK_{i}^{JPN} (t) = \max \left( {\frac{{L_{i}^{JPN} }}{{FA_{i}^{JPN} }},Y_{i}^{JPN\_Afp} } \right) \, t = 1 \hfill \\ OK_{i}^{JPN} (t) = \frac{{Yact_{i}^{JPN} (t)}}{{TFD_{i}^{JPN} (t)}} \, t > 1 \hfill \\ \end{gathered} \right. \, \left\{ {\begin{array}{*{20}c} {OK_{i}^{THA} (t) = \max \left( {\frac{{L_{i}^{THA} }}{{FA_{i}^{THA} }},Y_{i}^{THA\_Afp} } \right) \, t = N} \\ {OK_{i}^{THA} (t) = \frac{{Yact_{i}^{THA} (t)}}{{TFD_{i}^{THA} (t)}} \, t > N} \\ \end{array} } \right.$$

where $OK_{i}^{JPN} (1)$ and $OK_{i}^{THA} (N)$ refer to the initial shock of the Japan Earthquake / Thailand Flood on the *i*-th sector of Japan and Thailand. They are derived from the larger value of affected capital stocks (DEL $L_{i}^{JPN} /L_{i}^{THA}$ divided by capital stocks $FA_{i}^{JPN} /FA_{i}^{THA}$) and labor shortages ($Y_{i}^{JPN\_Afp} /Y_{i}^{THA\_Afp}$) on output. The calculation of the initial affected population and recovery curve are derived from the AMIL model (Zhang et al. 2019). The $OK_{i}^{JPN} (t > 1)$ and $OK_{i}^{THA} (t > N)$ represent the supply-demand imbalance during the post-recovery period of Japan and Thailand. The $Yact_{i}^{JPN} (t)$ and $Yact_{i}^{THA} (t)$ represent the actual production at the time *t* of the *i*-th sector of Japan and Thailand. The $TFD_{i}^{JPN} (t)$ and $TFD_{i}^{THA} (t)$ represent the actual total demand at the time *t* of the *i*-th sector after the disaster in Japan and Thailand.

In addition, the assessment of the labor shortages caused by JE and TF is based on the Basic Dynamic Inequalties (BDI) (Li et al. 2013) and the full-time equivalent approach (Hetrick 2000). The key equation is:

2 $$Y_{i}^{JPN\_Afp} = \frac{{\sum\limits_{k = 1}^{n} {Pop_{i}^{affeck - JPN,k} \cdot Time_{i}^{affect - JPN,k} } }}{{Pop_{i}^{total - JPN} \cdot FT_{i}^{JPN} }} \cdot \frac{{ICM_{i}^{JPN} }}{{X_{i}^{JPN} }}$$

where $Y_{i}^{JPN\_Afp}$ refers to the proportion of production reduction decided by the shortage of labor in the *i*-th sector due to the direct shock of JE; $ICM_{i}^{JPN}$ refers to *i*-th sector labor compensation in the initial input part of the input-output table, $X_{i}^{JPN}$ refers to the total output of the *i*-th sector of Japan; *k* means different types of impact on affected labor, including injury, missing people and death, emergency relocation, affected population, and so on (RIETI 2010; World Bank 2012; Tokui et al. 2017; Reconstruction Agency 2021); $Pop_{i}^{affeck - JPN,k}$ and $Time_{i}^{affect - JPN,k}$ represent the number and time of affected labor caused by JE in the *i*-th sector of the *k*-th type; $Pop_{i}^{total - JPN}$ and $FT_{i}^{JPN}$ represent the total number and yearly full working time of labors of the *i*-th sector of Japan. The equation and parameters for $Y_{i}^{THA\_Afp}$ stay the same.

Then the model is optimized for the impact of the disaster cluster on the global industrial chain both on the supply side and the demand side, based on the AMIL model (Zhang et al. 2019), respectively.

On the demand side, as supply decreases and demand increases at time* t* of the *i*-th sector after the disaster—if the surging reconstruction demand generated in Japan and Thailand cannot be met temporarily by domestic supply in the early post-disaster period—this part of the demand is set to be transferred to other countries/regions outside the disaster area to substitutable production through the global industrial chain (Eq. 3):

3 $$\left\{ {\begin{array}{*{20}c} {DT_{i}^{JPN} (t) = TFD_{i}^{JPN} (t) \cdot (1 - OK_{i}^{JPN} (t)) \, t > 1} \\ {DT_{i}^{THA} (t) = TFD_{i}^{THA} (t) \cdot (1 - OK_{i}^{THA} (t)) \, t > N} \\ \end{array} } \right.$$

where $DT_{i}^{JPN} (t)$ and $DT_{i}^{THA} (t)$ refer to the *i*-th sector of unmet reconstruction demands of Japan and Thailand. The demands are allocated to other countries/regions according to the closeness degree of the trade demand relationship between the countries/regions and Japan/Thailand under the delay of the trade overtime (Eq. 4), that is, the allocation is based on the ratio of each country’s total final demand to the total final demand of all countries/regions (except Japan/Thailand):

4 $$\left\{ {\begin{array}{*{20}c} {Dsub_{i}^{countryR} (t) = DT_{i}^{JPN} (t) \cdot \frac{{TFD_{i}^{R} }}{{\sum\limits_{A = 1}^{m(A \ne JPN)} {TFD_{i}^{A} (t)} }} \cdot \frac{1}{{\Delta Tr_{i}^{JPN \to R} }} \, 1 < t < N \, } \\ {Dsub_{i}^{countryR} (t) = DT_{i}^{JPN} (t) \cdot \frac{{TFD_{i}^{R} }}{{\sum\limits_{A = 1}^{m(A \ne JPN)} {TFD_{i}^{A} (t)} }} \cdot \frac{1}{{\Delta Tr_{i}^{JPN \to R} }} + DT_{i}^{THA} (t) \cdot \frac{{TFD_{i}^{R} }}{{\sum\limits_{A = 1}^{m(A \ne THA)} {TFD_{i}^{A} (t)} }} \cdot \frac{1}{{\Delta Tr_{i}^{THA \to R} }} \, t \ge N} \\ \end{array} } \right.$$

where $Dsub_{i}^{countryR} (t)$ is the unmet demand that the *R*-th country is to obtain temporarily as substitution production from Japan and Thailand; $\Delta Tr_{i}^{{JPN\mathop{\longrightarrow}\limits^{{}}{\text{R}}}}$ and $\Delta Tr_{i}^{{THA\mathop{\longrightarrow}\limits^{{}}R}}$ are the trade time (the median import and export time during shipment-at loading port-discharge port-consignee-receiver between countries/regions) from Japan and Thailand to the *R*-th country, obtained from the World Bank. When *1 < t < N*, country *R* receives the substitution production demand from Japan. When *t > N*, it will receive the demand from both Japan and Thailand. At this time, the total demand of the *i*-th sector of the *R-*th country is:

5 $$TFD_{i}^{countryR} (t) = TFD_{i}^{countryR} (t - 1) + Dsub_{i}^{countryR} (t)$$

where $TFD_{i}^{countryR} (t)$ is the total demand of the *i*-th sector at time *t*, $Dsub_{i}^{countryR} (t)$ is the substitution production demand received from Japan and Thailand, and this demand is composed of the domestic total demand $TFD_{i}^{countryR} (t - 1)$ at time *t*-1 of the *i*-th sector and the substitution production demand $Dsub_{i}^{countryR} (t)$ from Thailand (*t* > *N*) and Japan (*t* > 1) at time *t* of the *i*-th sector.

When the supply in Japan and Thailand meets the reconstruction demand, the demand for substitution production gradually decreases and eventually completely shifts back to domestic production.

On the supply side, the model calculates the ripple effect of the supply reduction of Japan and Thailand on the global industrial chain based on the multi-regional Leontief production function. The *i*-th sector of supply reduction of Japan and Thailand will affect the supply of other sectors of the *R*-th country outside the disaster-affected country through the direct consumption matrix $A_{i}^{other}$(Eq. 6):

6 $$\left\{ {\begin{array}{*{20}c} {Y_{i}^{R} (t) = \sum\limits_{i = 1}^{n} {(I - (1 - OK_{i}^{JPN} )A_{i}^{JPN \to R} ) \cdot TFD_{i}^{JPN \to R} } + \sum\limits_{i = 1}^{n} {(I - A_{i}^{other} )^{ - 1} \cdot TFD_{i}^{other} } \, 1 \, < t < N \, } \\ {Y_{i}^{R} (t) = \, \sum\limits_{i = 1}^{n} {(I - (1 - OK_{i}^{JPN} )A_{i}^{JPN \to R} ) \cdot TFD_{i}^{JPN \to R} } + \, \sum\limits_{i = 1}^{n} {(I - (1 - OK_{i}^{THA} )A_{i}^{THA \to R} ) \cdot TFD_{i}^{THA \to R} } + \sum\limits_{i = 1}^{n} {(I - A_{i}^{other} )^{ - 1} \cdot TFD_{i}^{other} } t \ge N} \\ \end{array} } \right.$$

where $(1 - OK_{i}^{JPN} )A_{i}^{JPN \to R}$ indicates the import reduction $OK_{i}^{JPN}$ from Japan received by the *i*-th sector in the *R*-th country; $(1 - OK_{i}^{THA} )A_{i}^{THA \to R}$ does so similarly but from Thailand; $A_{i}^{other}$ refers to the linkage between the *i*-th sector in the *R-*th country and the various sectors in other countries/regions. When 1 < *t* < N, the *i*-th sector of the production in the *R-*th country is only affected by the Japan Earthquake, the degree of industrial linkage decreases $OK_{i}^{JPN} (t)$. When *t* > N, the *i*-th sector of the production in the *R-*th country is influenced by both the Japan Earthquake and the Thailand Flood, the degree of industrial linkage decreases $OK_{i}^{JPN} (t)$ and $OK_{i}^{THA} (t)$, respectively.

In addition, the model also sets the following three modules: (1) The overproduction module is set to accelerate the recovery speed of production capacity in Japan and Thailand. This module depends on the post-disaster government rescue effort and insurance compensation; (2) The supply recovery module emphasizes the dynamic recovery process of labor and capital stocks, respectively. The labor recovery curve follows the hyperbolic tangent function based on Bruneau resilience theory (Bruneau et al. 2003), and the capital stock recovery uses overproduction parameters from the overproduction module. The recovery function is spatially heterogeneous and sector specific; (3) The production bottleneck module is set to judge whether the *i*-th sector of production capacity is affected by the related sector in other regions and evaluate the degree of impact.

The model adopts the balance of production capacity and total demand in Japan and Thailand as the basis for the end of post-disaster reconstruction. The IELs for Japan and Thailand and the EREs for other countries/regions are calculated by Eq. 7:

7 $$R\_loss = \sum\limits_{j = 1}^{p} {\sum\limits_{i = 1}^{q} {\sum\limits_{t = 1}^{n} {(VA_{i,j}^{pre} \times n - VA_{i,j}^{act} (t))} } }$$

where *R_loss* refers to the IELs and EREs for Japan, Thailand, and other countries/regions, *t* means time for post-disaster reconstruction (*n* days), *i* means sector (*q* sectors), and *p* means all the countries/regions (*p* countries); $VA_{i,j}^{pre}$ refers to the value added before the disaster occurred, $VA_{i,j}^{act} (t)$ refers to the value added at day *t* during the reconstruction period, calculated by Eq. 8:

8 $$VA_{i,j}^{act} (t) = VA_{i,j}^{pre} \cdot \frac{{Yact_{i}^{j} (t)}}{{Y_{i,j}^{pre} }}$$

where $Yact_{i}^{j} (t)$ represents the actual production after the disaster, and $Y_{i,j}^{pre}$ represents the production before the disaster.

In addition, due to the lack of IEL statistical data, the uncertainty analysis is carried out combined with the AMIL model (Zhang et al. 2018; Zhang et al. 2019) and the flood footprint model (Mendoza-Tinoco et al. 2017). The parameters used for the GERIO model are divided by standard and validation groups. The parameter values derived from actual statistics and data from the literature are set as the standard group, and the upper and lower limits of the uncertainty parameter value intervals are set as + 30% and − 30% of the standard parameter values, respectively. For the results of the uncertainty range for this study see Fig. 5.

This article shows the key optimization improvement of the GERIO model compared with the AMIL model. For a specific description of other relevant modules, equations, and parameters of the GERIO model, see the AMIL model (Zhang et al. 2018; Zhang et al. 2019).

## Evaluation Results and Analysis

This section analyzes the EREs caused by the disaster cluster to countries around the world, the difference between the cumulative EREs of the disaster cluster and the summation EREs caused by each disaster, and the key factors causing this difference.

### The Economic Ripple Effects (EREs) of the Disaster Cluster on the Economies of Countries/Regions Around the World

**Fig. 4:**
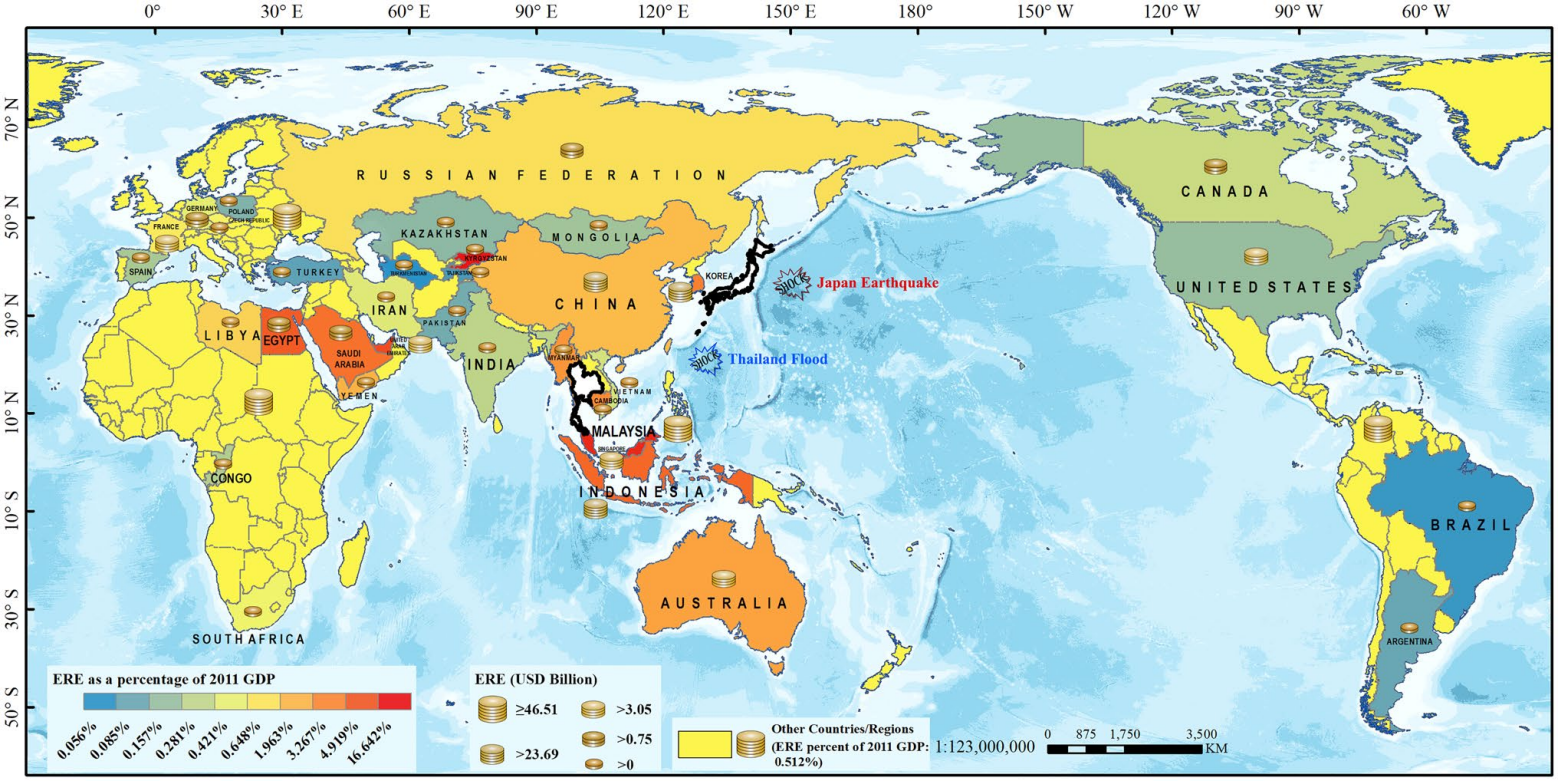
Global economic ripple effects (EREs) caused by the 2011 Japan/Thailand disaster cluster

The overall EREs caused by the disaster cluster (Scenario 3) reached USD 385.61Billion, 2.3 times the DEL. Malaysia, China, Indonesia, South Korea, and the United States were the top five countries with high ERE (Fig. 4). Malaysia suffered the highest ERE from the disaster cluster, reaching USD 46.51 Billion (excluding the RoW regions), which is the same as the DEL caused by the Thailand Flood.

Although China, South Korea, and France suffered a large absolute ERE, the impact on their GDP was relatively small. Saudi Arabia, Egypt, and Myanmar suffered a small absolute ERE, but the impact on their GDP was large (Fig. 4).

**Fig. 5:**
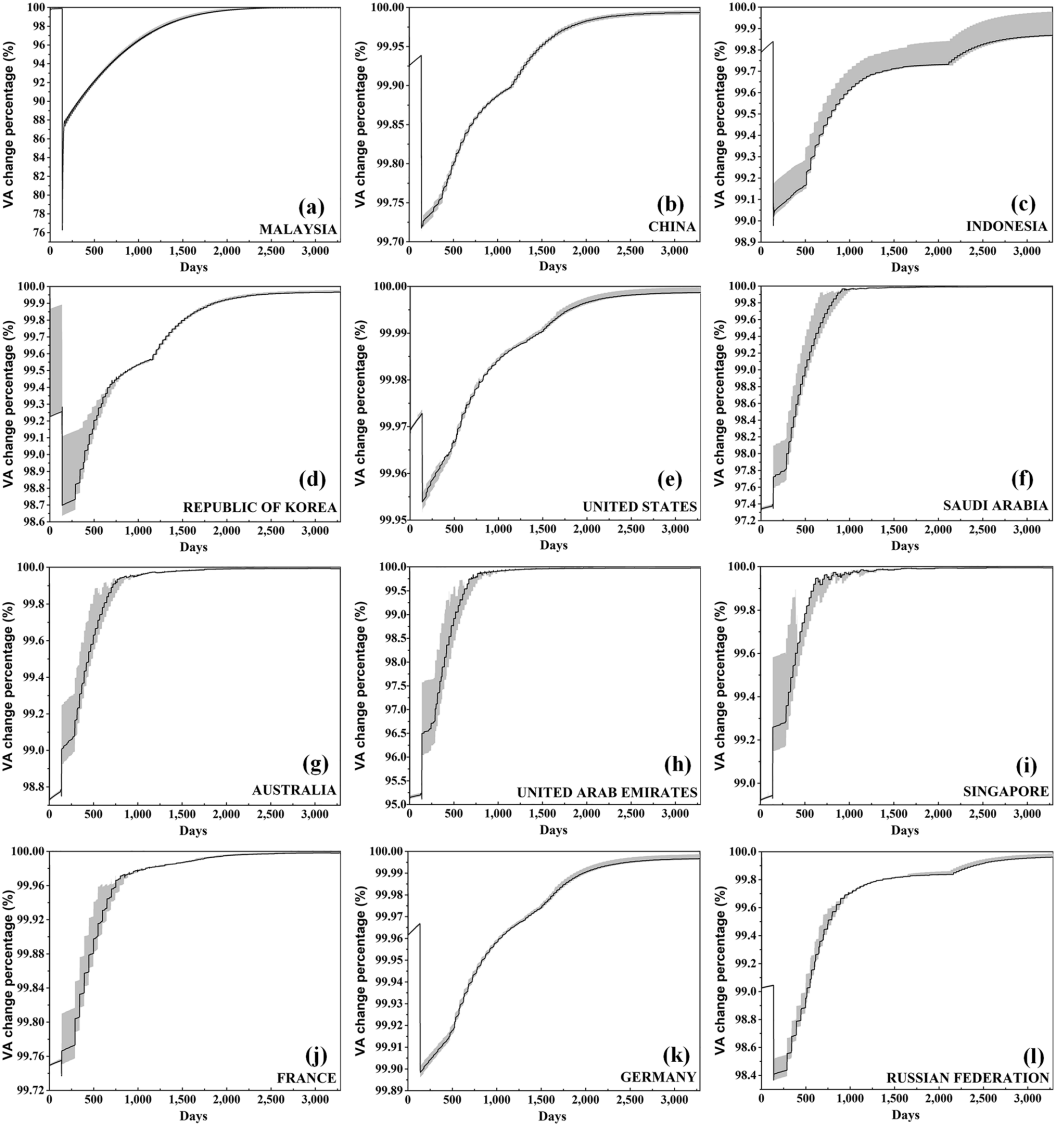
Recovery curve of the value added (VA) change rate due to the economic ripple effects (EREs) caused by the 2011 Japan/Thailand disaster cluster in the top 12 countries affected by EREs

For the dynamic ERE results (Fig. 5), the recovery curve of the value added (VA) change rate of the top 12 affected countries can be divided into two stages: the first is the period after the Japan Earthquake and before the Thailand Flood (11 March 2011−25 July 2011); the second is after the Thailand Flood (25 July 2011 onward). By comparing the recovery characteristics of countries in these two stages, the degree of GDP decline and the recovery effect are mainly determined by: (1) the trade distance between each country and the disaster-affected country; (2) the strength of the trade ties; and (3) the GDP development of each country.

**Fig. 6:**
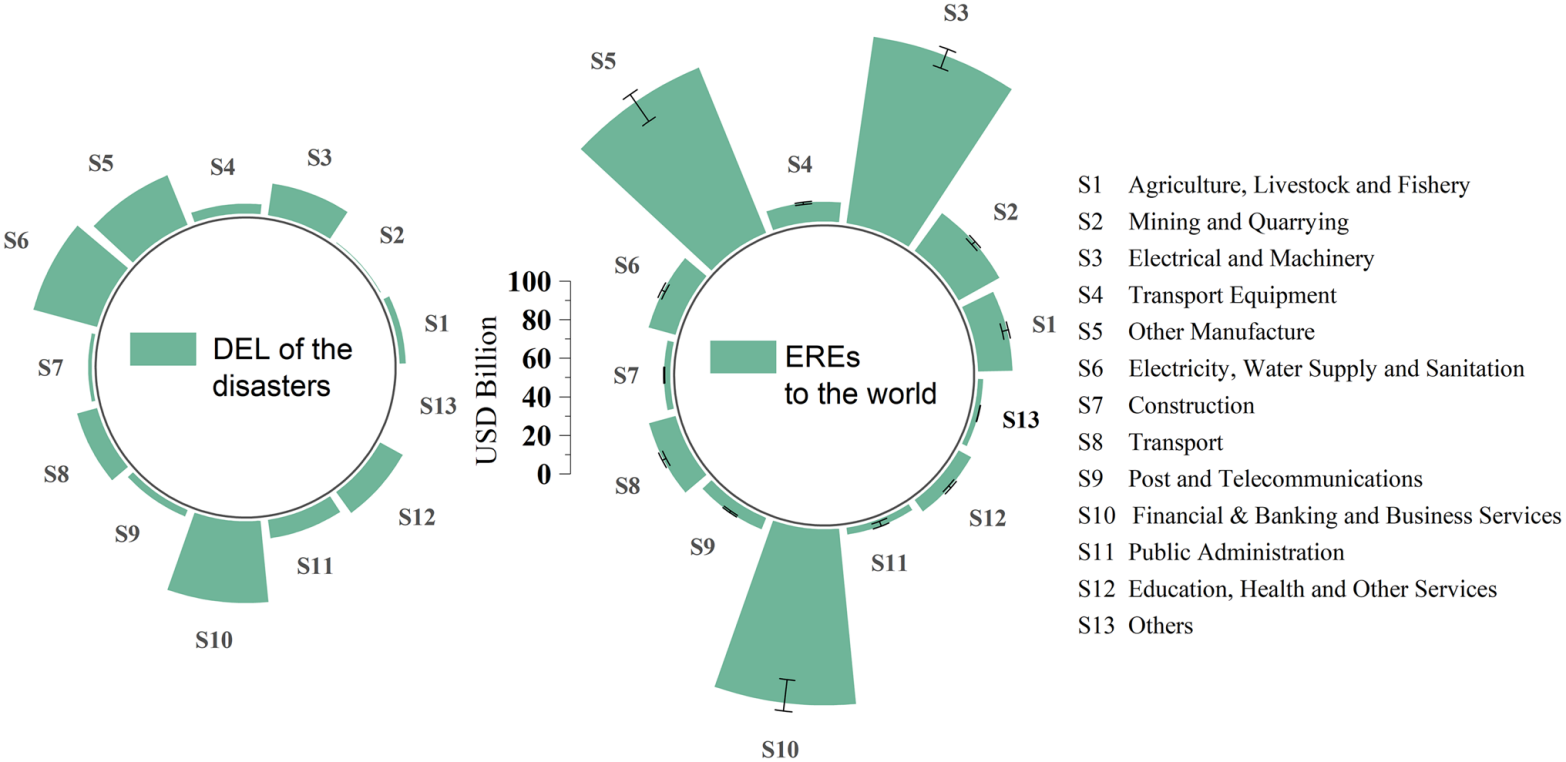
Comparison between the direct economic losses (DELs) and the economic ripple effects (EREs) of 13 sectors around the world caused by the 2011 Japan Earthquake (JE) and the 2011 Thailand Flood (TF) disaster cluster

The evaluation results at the sector scale show that the EREs have the amplification effect in sectors and the aggregation effect in key sectors (Fig. 6). If the affected sector has a stronger industrial influence in the global industrial chain, and this sector’s trade time is fast, the DEL suffered by the sector can usually further ripple to the countries around the world through the industrial chain, and the EREs are concentrated in key node sectors (electronic equipment manufacturing, other manufacturing, and business services in this study). However, for sectors with low transmission in the industrial chain, losses are mainly limited to the disaster country/region (such as the energy industry, public administration, and education industry), but these sectors may indirectly affect other sectors in the disaster area, and through them cause further EREs to other regions.

The above results highlight the importance of evaluating the EREs caused by the disaster cluster. They emphasize that when preventing major disaster economic risk, a country should not only improve its own disaster prevention and mitigation capabilities, but also pay attention to the economic ripple effects caused by a catastrophic disaster in other countries (especially those countries with close trade ties and neighboring countries).

### Difference Between the Cumulative Economic Ripple Effects (EREs) of the Disaster Cluster and the Summation EREs Caused by the Individual Disasters

Based on the assessment results, we analyzed the difference (hereafter referred to as the ERE difference) between the summation EREs caused by the two individual disasters (Scenarios 1 and 2) and the cumulative EREs of the disaster cluster (Scenario 3).

**Fig. 7:**
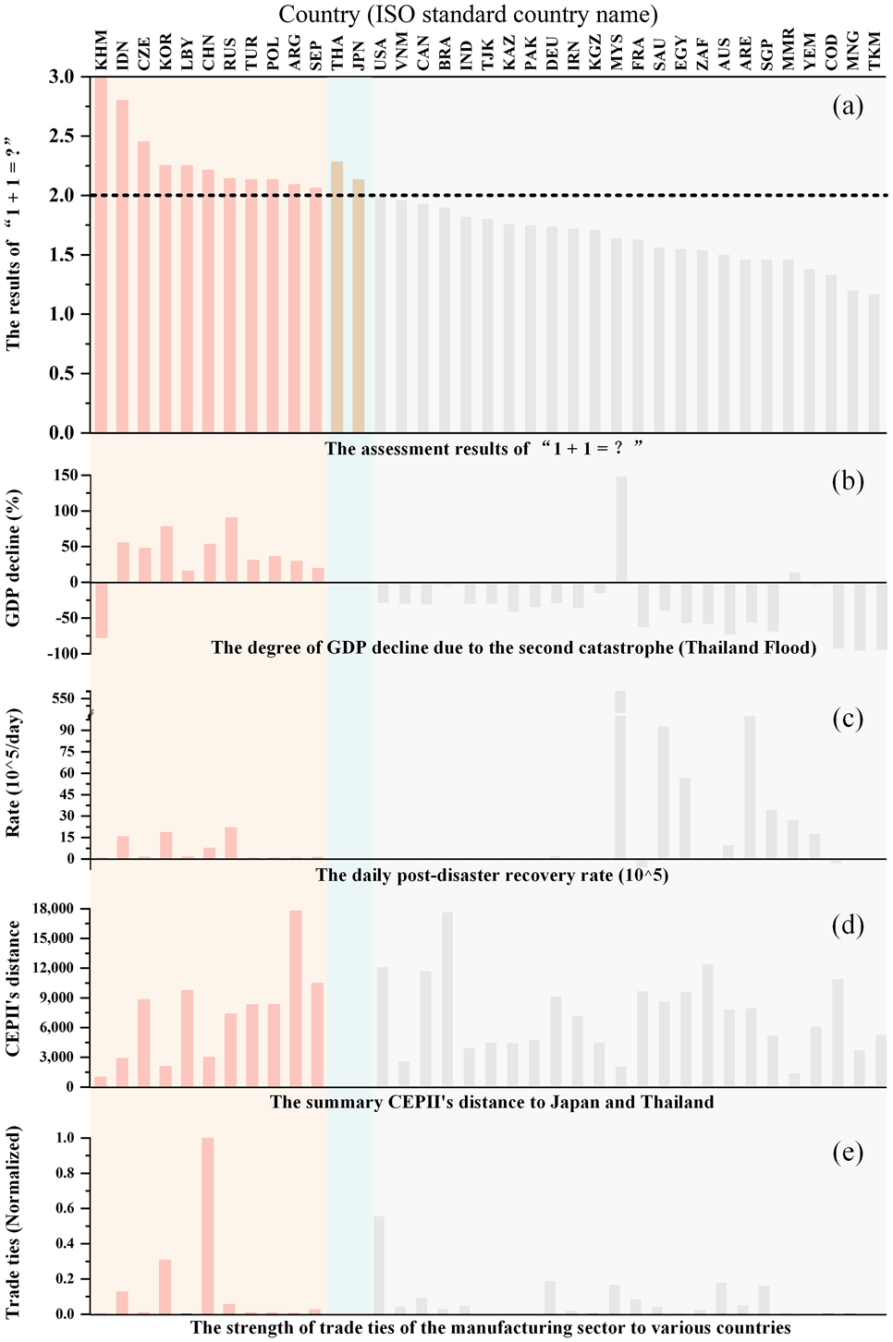
The assessment results of “1 + 1 = ?” and the characteristic analysis for “1 + 1 > 2.” **a** Refers to the results of the “1 + 1 = ?”; **b**−**e** Represent the characteristics between countries of “1 + 1 > 2” and “1 + 1 < 2.” Pink indicates countries with “1 + 1 > 2,” blue indicates Japan and Thailand, and grey indicates countries with “1 + 1 < 2.” **b** Refers to the degree of the second GDP decline when the countries/regions were affected by the second catastrophe (Thailand Flood); **c** Refers to the recovery rate per day in the whole recovery period, where we magnified the ratio (%) by 10^5^ for better display; **d** Refers to the sum of the Centre d’Études Prospectives et d’Informations Internationales (CEPII)’s distance between countries and Japan/Thailand (Mayer and Zignago 2011); **e** Refers to the strength of trade ties composed of pushing and pulling forces.

For Japan and Thailand, the total indirect economic impact the two countries suffered is not only the indirect economic loss (IEL) of their own country’s catastrophe, but also the ERE of the other catastrophe to their country. In Scenario 1, the IEL caused by the Japan Earthquake to Japan was USD 135.71Billion, and the ERE to Thailand was USD 2.11 Billion. In Scenario 2, the IEL caused by the Thailand Flood to Thailand was USD 119.84 Billion, and the ERE to Japan was USD 22.81 Billion. Therefore, Japan suffered a total indirect impact of USD 158.52 Billion from the two catastrophes, while Thailand suffered a total indirect impact of USD 121.95 Billion. However, in the Scenario 3 disaster cluster, Japan suffered a total indirect impact of USD 179.18 Billion, and Thailand suffered USD 155.68 Billion, both of which are greater than the summation loss caused by the two individual catastrophes. Therefore, the results are “1 + 1 = (1 + 179.18/158.52) = 2.13” and “1 + 1 = (1 + 155.68/121.95) = 2.28” for Japan and Thailand, respectively, that is, “1 + 1 > 2” (Fig. 7a). This result indicates that in the post-disaster recovery and reconstruction period, the disaster-affected country should pay more attention to and prevent the secondary external shocks caused by catastrophes outside the region, and adopt government regulations and market adjustment measures that target the emergency replacement of post-disaster recovery products and services, so as to reduce the amplification effect of external shocks on the regional economic system.

For the countries/regions outside Japan and Thailand, the ERE difference is significant between them. Among the other 35 countries/regions (not including Japan, Thailand, and the rest of the world), the cumulative EREs caused by the disaster cluster (Scenario 3) in 11 countries are greater than the summation EREs caused by the two individual catastrophes (Scenario 1 + Scenario 2)—for example, “1 + 1 = 2.21” in China, “1 + 1 = 2.80” in Indonesia, “1 + 1 = 2.25” in South Korea, and so on (Fig. 7a).

It can be concluded that the four major differences between the countries with “1 + 1 > 2” and “1 + 1 < 2” are: (1) When the economic system has not recovered from the first catastrophe, the degree of second GDP decline of the “1 + 1 < 2” countries is lower than that of the “1 + 1 > 2” countries (see Fig. 7b); (2) The recovery rate of the “1 + 1 < 2” countries in the second recovery stage is higher than that of the “1 + 1 > 2” countries (see Fig. 7c); (3) The difference of the “1 + 1” results has a certain relationship with the trade distance between the affected countries and other countries—for example, for Cambodia (KHM), Indonesia (IDN), and China CHN), their close trade distances are the main factor contributing to the result of “1 + 1” value higher than 2, but it does not apply to all the countries (see Fig. 7d); (4) The difference of the “1 + 1” results also has a certain relationship with the strength of trade ties—for example, for Indonesia (IDN), South Korea (KOR), and China (CHN), their strong trade ties with Japan and Thailand are the main factor contributing to the result of “1 + 1” value higher than 2. This relationship also does not apply to all the countries (see Fig. 7e).

In the above “1+1 is greater or less than 2” difference analysis, the evaluation results of “1 + 1 > 2” is easier to understand than the results of “1 + 1 < 2.” For the countries with the “1 + 1 < 2” results, their relationship with the country affected by the second disaster (Thailand) was the main reason for these results. For example, Brazil (BRA) and Canada (CAN) have a large trade distance to Thailand (see Fig. 7d) and a low industrial linkage to Thailand’s manufacturing industry (see Fig. 7e), therefore they were less susceptible to the Thailand Flood and may have been able to take on some substitutional production demand from Thailand; Malaysia (MYS) has a close trade distance (see Fig. 7d) and close industrial linkage to Thailand (see Fig. 7e) and its economic system was more affected by the Thailand Flood initial shock than that of the Japan Earthquake (see Fig. 7b), so the government and enterprises attached great importance to the Thailand Flood and responded in a timely manner, resulting in a fast recovery rate (see Fig. 7c); The United Arab Emirates (ARE) has a long trade distance (see Fig. 7d) and low industrial linkage (see Fig. 7e) but due to the uniqueness of some products like petrochemicals in the global industrial chain, the reconstruction of the disaster-affected country increased the demand for these products and resulted in positive impacts on petroleum extraction and processing, which became part of the reason for the “1 + 1 < 2” result in the country.

Therefore, the difference of the “1 + 1” results is the result of a combination of many factors, which mainly include the trade distance, the strength of trade ties between other countries/regions and the disaster-affected countries, the economic resilience of individual countries/regions, as well as the industrial influence of affected sectors in the disaster-affected countries on sectors of all countries/regions.

### Analysis of the Key Factors Causing the Difference between the Cumulative and Summation Economic Ripple Effects (EREs)

According to the ERE assessment results and the ERE difference analysis, two easily forgotten factors in the results of the ERE difference can be further summarized:

- (1) The industrial dependence of EREs may be one of the key factors causing the ERE difference among countries/regions. As the transmission routes of EREs are mostly concentrated in key sectors with strong industrial influence and fast trade times in the industrial chain (such as manufacturing), one of the important reasons for the ERE difference is industrial influence. Therefore, the closer the trade ties between a country and the disaster-affected country in key sectors, the more significant the impact of the EREs on the country’s industrial chain will be, and the more likely it is that the country will belong to the “1 + 1 > 2” country group.
- (2) The extension of the post-disaster recovery period of the affected countries due to the disaster cluster may be another key factor causing the ERE difference among countries/regions. Since the economic system of the disaster-affected country has not fully recovered after the first catastrophe, the recovery period of that country after the second catastrophe will be longer than that of the country suffering from only the second catastrophe. During the extended recovery period, the country may face more EREs caused by new catastrophes in other countries/regions around the world, and may also cause greater EREs to the global industrial chain in combination with new catastrophes in other countries/regions, while the extra EREs are suffered more by countries/regions that have strong trade ties with the disaster-affected country.

**Fig. 8:**
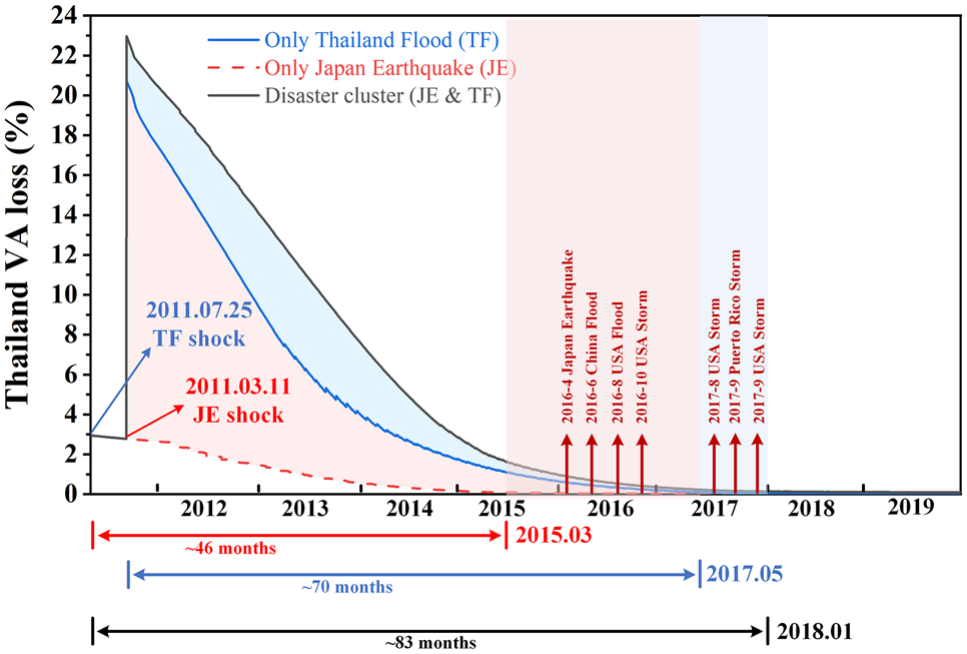
Comparison of recovery period from 2011 Thailand Flood under three scenarios in Thailand. In order to highlight the comparative effect of the impact of a disaster cluster on the extension of the recovery period under the three scenarios, we multiplied the value of value added (VA) loss in Thailand within 0−136 days by 10, which did not change the recovery period and is only applied to the display of the figure. The blue shade represents the additional loss under Scenario 3 compared with Scenario 2, and the pink shade represents the additional loss under Scenario 3 compared with Scenario 1

Take the recovery period of Thailand under the three disaster scenarios as an example (Fig. 8). When only the Japan earthquake occurred (Scenario 1), Thailand’s GDP would have been restored by March 2015 with a recovery period of 46 months. When only the Thailand Flood occurred (Scenario 2), Thailand’s GDP would have been restored by May 2017 with a recovery period of 70 months. However, when the actual disaster cluster occurred (Scenario 3), Thailand’s recovery period was extended to January 2018, with a recovery period of 83 months, 13 months longer than that of Scenario 2, and 37 months longer than that of Scenario 1. In addition, the losses Thailand suffered were 1.43 times those of Scenario 2, and 78 times those of Scenario 1. These additional losses are more likely to be transmitted to countries/regions with close trade ties to Thailand, further expanding the difference between the cumulative loss and the summation loss. Furthermore, during the extended recovery period, seven new catastrophes with DELs of more than USD one billion occurred globally. Therefore, Thailand’s economic system may suffer more EREs, and may form a new disaster cluster with these new catastrophes to cause greater loss in the global industrial chain.

Therefore, with the increasing frequency of disaster clusters in the future, countries/regions should pay attention to the trade distance and the strength of trade ties with the countries where a catastrophic disaster has occurred, especially with those where the second catastrophe has occurred. If a country has a short trade distance and strong trade ties with the disaster-affected country, it should focus on preventing the cumulative EREs caused by a disaster cluster, and reduce the possible economic impact through enforcing targeted government regulations and market adjustment measures.

## Discussion

Although the differences between the cumulative EREs of the disaster cluster and the summation EREs separately caused by the catastrophes and the main reasons behind them are analyzed, there are still some gaps in this study that need to be explored in further research:

- (1) Thailand’s manufacturing industry has a great impact on the global supply chain of automobiles and electronic equipment, which is also reflected in the prices of automobiles and hard disks. However, this study macroscopically assumed that the price is affected by the ratio of supply and demand and a price response parameter, not considering other characteristics such as cost and market. So the price can only play a role to a certain extent although it does not invalidate the price assumption of the input-output model. Meanwhile, as the macro loss assessment cannot accurately depict the micro industry price fluctuations, it needs a large number of exogenous variables to accurately calculate. This is also an important topic for our future research.
- (2) The advantage of the GERIO model is that it considers the impact of substitution production for unmet recovery demand on reducing EREs, but its weakness is that it is still difficult to calculate the impact of inter-sector substitution production on decreasing IEL due to the nature of rigid inter-industry connections in the input-output model. Based on the theory of the Multiregional Impact Assessment (MRIA) model (Koks and Thissen 2016), inter-sector substitution will be the major improvement in future research.
- (3) The timestep of the GERIO model is “day” but the world input-output table is calculated by year. The GERIO model homogenizes the input-output table by day as most other studies, but it is still difficult to reflect the difference in the daily industrial linkage of each country (Avelino 2017) (for example, the IEL and ERE of the agriculture sector will be smaller if it suffers the shock after the harvest period than during the growing period), so this limitation will be optimized from the perspective of data and modeling in the future.

In addition, the occurrence of a disaster cluster cannot be foreseen due to a high degree of temporal and spatial variability. When developing regional industrial resilience, we should: (1) reversely evaluate the related regions through the supply chain of the prime industries in a region, and understand the disaster risk in these related regions, guaranteeing a certain substitutional supply capacity of raw material products when the impact of a disaster ripples to the region. The purpose is to enhance the substitutability of upstream and downstream products of regional prime industries; (2) understand the trade routes (land, shipping, air, and so on) with the related regions, and evaluate the risk of disaster to trade routes. The purpose is to enhance the transportation resilience of regional industries through rational industrial layout and improvement of trade routes; and (3) expand the scope of disaster insurance and enhance the awareness of the EREs of a disaster cluster. The purpose is to improve the risk prevention awareness of regional industries and formulate relevant market response measures. In addition, it is necessary to take risk prevention measures according to local conditions by considering the geographic location, regional development orientation, and economic development level of different regions, so as to curb the potential impact of a catastrophic cluster.

## Conclusion

This study used the GERIO model to evaluate the IELs and EREs caused by a disaster cluster that occurred in a very short time interval in two countries, and the EREs to 35 countries/regions around the world. The results show that:

1. The cumulative effect of the EREs in selected countries/regions caused by a disaster cluster is not equal to the summation EREs caused by the two disasters individually, and the difference is significant. In this study, 30% of the 35 countries/regions suffered higher cumulative EREs as compared to the summation EREs, while the other 70% suffered lower cumulative EREs than the summation EREs. The main reasons for this difference are a combination of many factors, including the economic influence of the disaster-affected sectors in the industrial chain of various countries/regions, the economic resilience of individual countries/regions, the trade distance, and the strength of trade ties between other countries/regions and the disaster-affected country.
2. Compared with DEL, the EREs caused by a disaster cluster have the amplification effect and industrial aggregation, which is also an important factor leading to the ERE difference. When the economic system is affected by the second catastrophic disaster without recovering from the first one, the amplification effect of a disaster cluster is significant. In this study, the EREs caused by the disaster cluster were more than two times that of the DELs caused by the two individual disasters. Countries/regions with a close trade distance to the two disaster-affected countries, such as Malaysia, China, and South Korea, as well as countries/regions with close trade ties with the disaster-affected countries, such as the United States, Saudi Arabia, and Australia, were more severely affected. In terms of sector distribution, the key sectors with high influence and fast trade times in the industrial chain such as manufacturing may suffer the most from EREs.
3. The extension of the post-disaster recovery period in disaster-affected countries caused by a disaster cluster may cause more EREs to affect the global industrial chain, and the additional losses during the extended period are mostly suffered by countries/regions with strong trade ties to the disaster-affected countries, further magnifying the ERE difference. The extension of the recovery period may lead to more EREs caused by new catastrophic disasters in other countries/regions and create more chances to form a new disaster cluster with other disasters, thus causing greater losses to the global industrial chain.

This study quantitatively emphasized the importance of EREs caused by a disaster cluster and optimized the theoretical basis of the ERE assessment of disaster clusters. It also found that the industrial aggregation and the extension of the recovery period are the key factors that lead to the ERE difference. The study revealed that when a country is preventing major economic risks of disasters, it should not only improve its own capability of disaster prevention and mitigation, but also pay attention to the ripple impact caused by disasters from neighboring countries/regions and those with close trade ties.
